# CD45^+^CD33^low^CD11b^dim^ myeloid-derived suppressor cells suppress CD8^+^ T cell activity via the IL-6/IL-8-arginase I axis in human gastric cancer

**DOI:** 10.1038/s41419-018-0803-7

**Published:** 2018-07-09

**Authors:** Fang-yuan Mao, Yong-liang Zhao, Yi-pin Lv, Yong-sheng Teng, Hui Kong, Yu-gang Liu, Xiao-long Wu, Chuan-jie Hao, Weisan Chen, Mu-bing Duan, Bin Han, Qiang Ma, Ting-ting Wang, Liu-sheng Peng, Jin-yu Zhang, Ping Cheng, Chong-yu Su, Xiao-long Fu, Quan-ming Zou, Gang Guo, Xiao-lan Guo, Yuan Zhuang

**Affiliations:** 1 National Engineering Research Centre of Immunological Products, Department of Microbiology and Biochemical Pharmacy, College of Pharmacy, Third Military Medical University, Chongqing, China; 2 Department of General Surgery and Centre of Minimal Invasive Gastrointestinal Surgery, Southwest Hospital, Third Military Medical University, Chongqing, China; 3 La Trobe Institute of Molecular Science, School of Molecular Science, La Trobe University, Bundoora, VIC 3086 Australia; 4 Department of Pharmacy, Affiliated Hospital of North Sichuan Medical College, Nanchong, Sichuan Province China

## Abstract

Myeloid-derived suppressor cells (MDSCs) are a prominent component of the pro-tumoral response. The phenotype of and mechanisms used by MDSCs is heterogeneous and requires more precise characterization in gastric cancer (GC) patients. Here, we have identified a novel subset of CD45^+^CD33^low^CD11b^dim^ MDSCs in the peripheral blood of GC patients compared to healthy individuals. CD45^+^CD33^low^CD11b^dim^ MDSCs morphologically resembled neutrophils and expressed high levels of the neutrophil marker CD66b. Circulating CD45^+^CD33^low^CD11b^dim^ MDSCs effectively suppressed CD8^+^ T cells activity through the inhibition of CD8^+^ T cell proliferation and interferon-γ (IFN-γ) and granzyme B (GrB) production. The proportion of CD45^+^CD33^low^CD11b^dim^ MDSCs also negatively correlated with the proportion of IFN-γ^+^CD8^+^ T cell in the peripheral blood of GC patients. GC patient serum-derived IL-6 and IL-8 activated and induced CD45^+^CD33^low^CD11b^dim^ MDSCs to express arginase I via the PI3K-AKT signaling pathway. This pathway contributed to CD8^+^ T cell suppression as it was partially rescued by the blockade of the IL-6/IL-8-arginase I axis. Peripheral blood CD45^+^CD33^low^CD11b^dim^ MDSCs, as well as IL-6, IL-8, and arginase I serum levels, positively correlated with GC progression and negatively correlated with overall patient survival. Altogether, our results highlight that a subset of neutrophilic CD45^+^CD33^low^CD11b^dim^ MDSCs is functionally immunosuppressive and activated via the IL-6/IL-8-arginase I axis in GC patients.

## Introduction

Gastric cancer (GC) is the fourth most common cancer worldwide. GC patients frequently present with advanced stage disease, which has a poor prognosis and low survival rate^1^. The immune system of cancer patients is often perturbed by pro-tumorigenic signals from the tumor microenvironment. Counter to this, natural killer cells and T cells act as a critical component of anti-tumor immunity, in particular tumor-specific effector CD8^+^ T cells, which directly induce tumor cell cytotoxicity. Effector CD8^+^ T cell activity, however, is inhibited during the development and metastatic progression of GC^2^. This effect may still be amenable to immunomodulation, however, as tumor-specific CD8^+^ T cells from the peripheral blood of GC patients can still exert cytotoxicity following stimulation by peptide-pulsed cells in vitro^3^. Understanding the factors driving CD8^+^ T cell suppression is therefore critical for the most effective clinical modulation of anti-tumor immunity.

Immunosuppressive myeloid cells were first described in the 1980s in cancer patients^4^. A large body of evidence now exists on their immunosuppressive effects during cancer progression, with emphasis on their heterogeneous phenotypes and mechanisms of action. In humans, myeloid-derived suppressor cells (MDSCs) are broadly classified as either neutrophilic or MO MDSCs, and are phenotypically identified as being CD11b^+^CD15^+^CD66b^+^CD33^+^CD14^−^ or CD11b^+^CD15^−^CD33^+^CD14^+^HLA-DR^-/low^, respectively^5–8^. In our previous studies, we observed a subset of immunosuppressive myeloid cells in the peripheral blood of GC patients. This myeloid subset was CD66^+^ but CD33^low^CD11b^dim^ in surface phenotype, rather than being typically CD11b^+^CD33^+^. A negative correlation was also observed between the proportions of CD33^low^CD11b^dim^ myeloid cells versus CD8^+^ T cells in the peripheral blood of GC patients. We thus hypothesized that the GC-selective CD33^low^CD11b^dim^ myeloid subset identified might function as MDSCs, and thereby detrimentally influence the progression of GC.

MDSCs are recruited by pro-inflammatory signals from the tumor microenvironment and exert their immunosuppressive activities through the upregulation of arginase I, iNOS, indoleamine 2, 3 deoxygenase (IDO), nitric oxide (NO), and reactive oxygen species (ROS)^9,10^. Arginase I is a highly conserved enzyme that metabolizes host L-arginine^11^ from the extracellular environment, results in decreased expression of the TCRζ-chain of CD3, and then impaired proliferation and cytokine production of T lymphocytes^12^. Human neutrophilic MDSCs are known to upregulate arginase I to inhibit CD8^+^ T cell activity^13^, while pro-inflammatory cytokine such as IL-6 and IL-8 were reported to regulate the expression or exocytosis of arginase I^14,15^. We then hypothesized that the CD45^+^CD33^low^CD11b^dim^ myeloid subset function as suppressive cells through arginase I and regulated by these pro-inflammatory factors.

In this study, we further characterized the prevalence, phenotype, and function of CD45^+^CD33^low^CD11b^dim^ MDSCs identified in peripheral blood of GC patients. We found that the CD45^+^CD33^low^CD11b^dim^ MDSCs exhibited a CD66b neutrophilic phenotype, and that increased frequencies correlated with tumor stage and decreased overall survival in GC patients. We further demonstrated that this subset suppressed CD8^+^ T cells IFN-γ and granzyme B production via IL-6-induced and/or IL-8-induced arginase I production. Suppression of CD8^+^ T cell activity could be partially rescued upon blockade of the IL-6/IL-8-arginase I axis. In conclusion, CD8^+^ T cell-mediated immunotherapy in GC patients may require the modulation of suppressive CD45^+^CD33^low^CD11b^dim^ MDSCs to be maximally effective, in particular through the blockade of the IL-6/IL-8-arginase I axis.

## Results

### Neutrophilic CD45^+^CD33^low^CD11b^dim^ myeloid cells are increased in the peripheral blood of GC patients

**Fig. 1:**
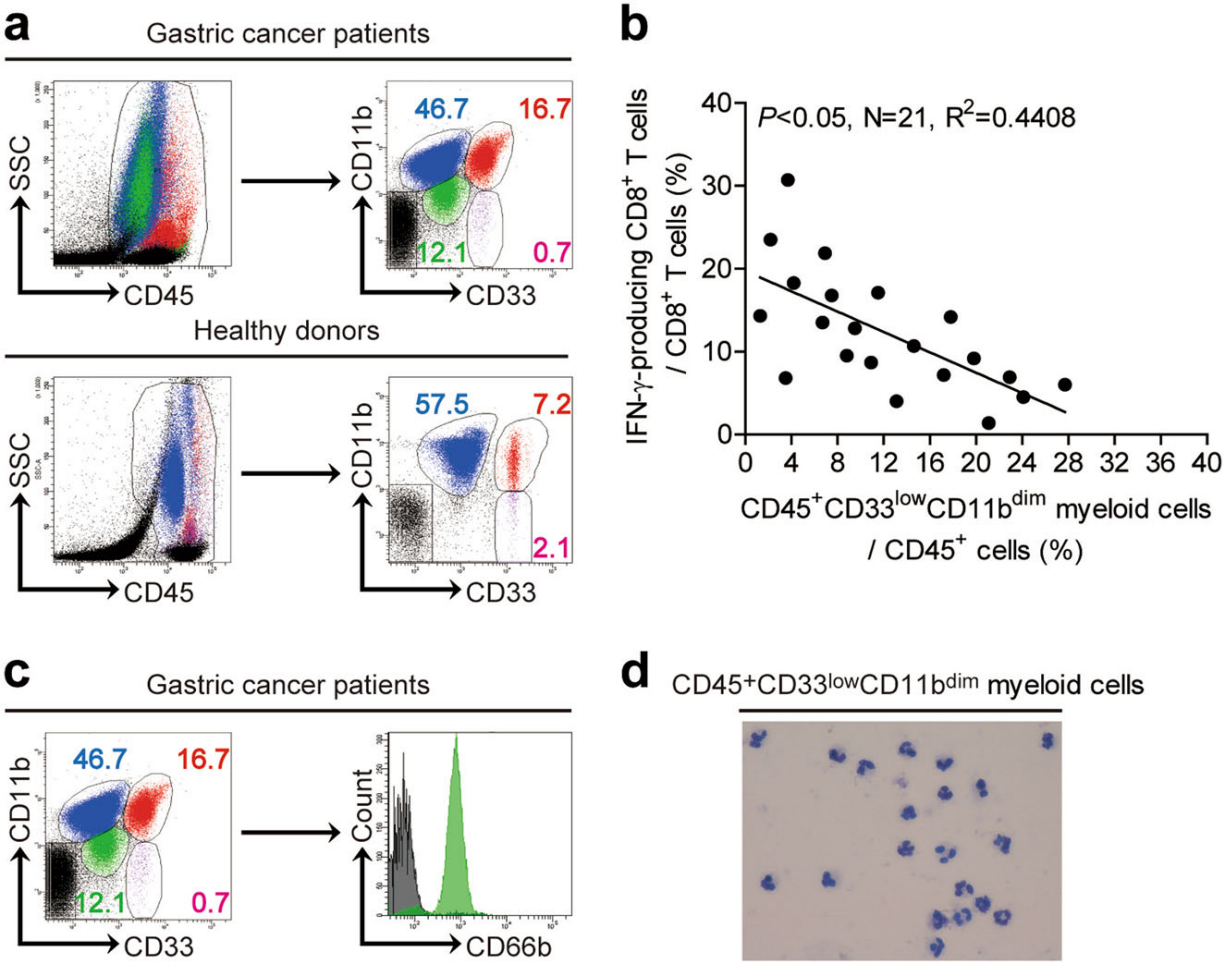
Peripheral blood CD45^+^CD33^low^CD11b^dim^ myeloid cells with a neutrophilic morphology are increased in patients with GC. **a** Surface expression of CD33 versus CD11b on CD45^+^ gated cells from the peripheral blood of GC patients or healthy donors as analyzed by flow cytometry. **b** Correlation between the percentages of peripheral blood CD45^+^CD33^low^CD11b^dim^ myeloid cell and IFN-γ-producing CD8^+^ T cells in GC patients. **c** Neutrophil marker CD66b surface expression on CD45^+^ gated CD33^low^CD11b^dim^ myeloid cells from the peripheral blood of GC patients. **d** Wright staining of sorted peripheral blood CD45^+^CD33^low^CD11b^dim^ myeloid cells from a GC patient. (red: CD45^+^CD33^+^CD11b^high^ cell; blue: CD45^+^CD33^low^CD11b^high^ cell; green: CD45^+^CD33^low^CD11b^dim^ cell)

We first confirmed that CD45^+^CD33^+^CD11b^+^ myeloid cells were significantly increased in the peripheral blood of GC patients compared to healthy donors, in concordance with other reports^16,17^. Interestingly, we also identified a myeloid cell subset, which was CD45^+^CD33^low^CD11b^dim^ and uniquely appeared in the peripheral blood of GC patients compared to healthy donors (Fig. 1a). As MDSCs are frequently found in cancer patients, we analyzed for the potential correlation between the frequency of CD45^+^CD33^low^CD11b^dim^ myeloid cells and IFN-γ-producing CD8^+^ T cells in GC patients, and found it to be significantly inversed (Fig. 1b). We further characterized this CD45^+^CD33^low^CD11b^dim^ myeloid cell subset using known human neutrophilic or MO MDSC surface markers, and found that they expressed high levels of the neutrophil marker CD66b and exhibited a multi-lobar nucleus characteristic of neutrophils (Fig. 1c, d). We also examined the CD45^+^CD33^low^CD11b^high^ myeloid cells subset, which also highly expressed CD66b (Supplementary Figure 1). We thus identified a novel GC patient selective subset of neutrophilic CD45^+^CD33^low^CD11b^dim^ myeloid cells in peripheral blood.

### Peripheral blood CD45^+^CD33^low^CD11b^dim^ myeloid cells suppress CD8^+^ T cell activity in vitro

**Fig. 2:**
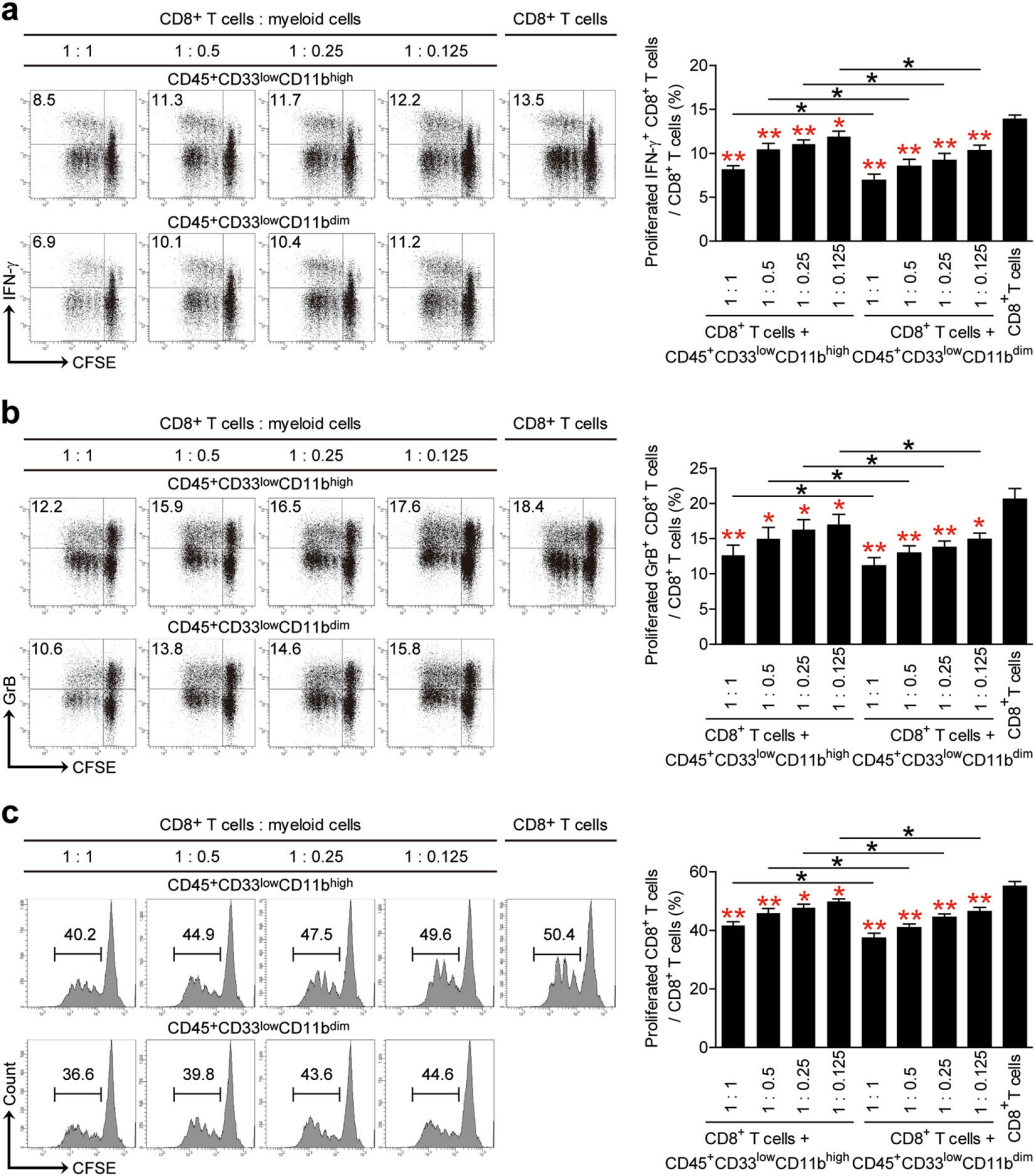
Peripheral blood CD45^+^CD33^low^CD11b^dim^ myeloid cells suppress CD8^+^ T cell activity. CFSE-labeled peripheral CD8^+^ T cells from healthy donors were co-cultured for 5 days with CD45^+^CD33^low^CD11b^high^ or CD45^+^CD33^low^CD11b^dim^ myeloid cells from autologous GC patients at different ratios. **a** CD8^+^ T cell proliferation, **b** IFN-γ and **c** granzyme B production was quantified by flow cytometry with data representative of three independent experiments (*n* = 3). *p < 0.05; **p < 0.01, and n.s, *p* > 0.05 for comparisons between respective groups (black) or compared with CD8^+^T cell control (red)

To evaluate the function of the CD45^+^CD33^low^CD11b^dim^ and also CD45^+^CD33^low^CD11b^high^ myeloid cell subsets, we sorted them from the peripheral blood of GC patients by fluorescence-activated cell sorting (FACS) and then co-cultured them with purified CD8^+^ T cells at different ratios for 5 days. We used typical regulatory T cells as positive control (data not shown), and found that both myeloid cell subsets suppressed CD8^+^ T cell proliferation and IFN-γ and GrB production upon allogeneic antigen stimulation (Fig. 2a–c). CD45^+^CD33^low^CD11b^dim^ myeloid cells were marginally more suppressive of CD8^+^ T cell activity compared to CD45^+^CD33^low^CD11b^high^ myeloid cells (Fig. 2a–c). Furthermore, CD45^+^CD33^low^CD11b^dim^ cell frequency positively correlated with patient tumor size and stage (Supplementary Figure 2). Altogether, these findings suggested that CD45^+^CD33^low^CD11b^dim^ myeloid cells behaved as MDSCs.

### CD45^+^CD33^low^CD11b^dim^ myeloid cells suppress CD8^+^ T cell activity via arginase I production

**Fig. 3:**
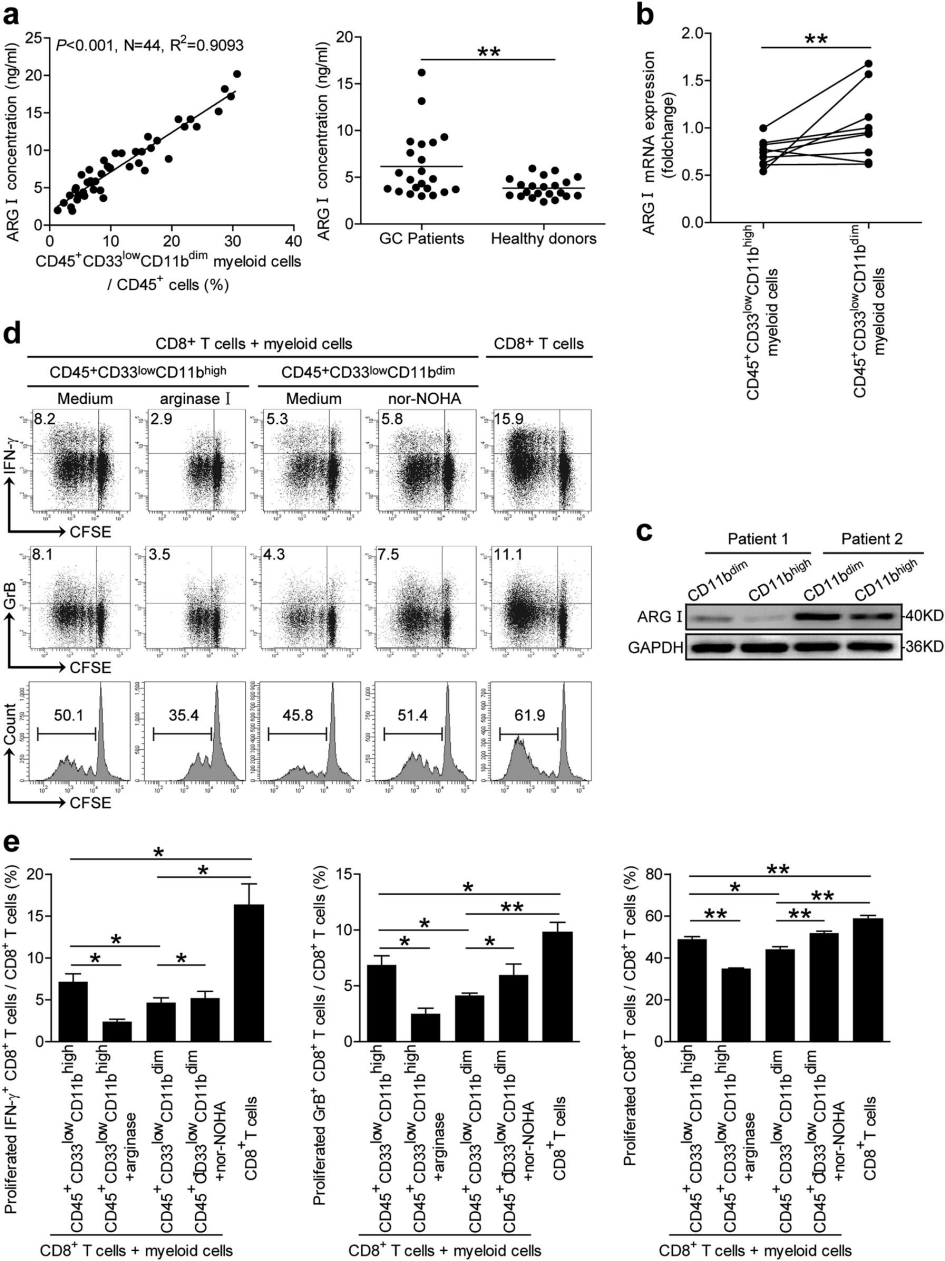
Peripheral blood CD45^+^CD33^low^CD11b^dim^ myeloid cells suppress CD8^+^T cell function via arginase I (ARG I) production. **a** The correlation between the percentage of peripheral blood CD45^+^CD33^low^CD11b^dim^ myeloid cells and the serum concentration of arginase I was analyzed in GC patients. Serum arginase I concentration was also analyzed in GC patients compared to healthy donors. **b**, **c** Arginase I expression in CD45^+^CD33^low^CD11b^high^ myeloid cells and CD45^+^CD33^low^CD11b^dim^ myeloid cells by quantitative real-time PCR and western blot, respectively. **d**, **e** CFSE-labeled peripheral CD8^+^ T cells from healthy donors were co-cultured for 5 days with either autologous peripheral blood CD45^+^CD33^low^CD11b^high^ myeloid cells from GC patients in the presence or absence of arginase I, or autologous CD45^+^CD33^low^CD11b^dim^ myeloid cells from GC patients in the presence or absence of nor-NOHA. CD8^+^ T cell proliferation and IFN-γ and granzyme B production experiments are representative of three independent experiments (*n* = 3). *p < 0.05; **p < 0.01, and n.s, *p* > 0.05 between respective groups. ARG I arginase I

CD8^+^ T cell suppression can be mediated via cell-to-cell contact (i.e., engagement of checkpoint receptors) or soluble factor release (i.e., ROS, NO, and arginase I). Human neutrophilic MDSCs are known to upregulate arginase I to inhibit CD8^+^ T cell activity^13^. We thus investigated whether CD8^+^ T cell suppression was dependent on arginase I activity in CD45^+^CD33^low^CD11b^dim^ myeloid cells. The concentration of serum arginase I was increased in GC patients compared to healthy donors (Fig. 3a); and a positive correlation was found between serum arginase I concentration and the frequency of peripheral blood CD45^+^CD33^low^CD11b^dim^ myeloid cells (Fig. 3a). We then investigated the capacity of arginase I production by two myeloid subsets, and found that the CD45^+^CD33^low^CD11b^dim^ myeloid cells produce more arginase I both in mRNA and protein levels, when compared to the CD45^+^CD33^low^CD11b^high^ myeloid cells (Fig. 3b, c). To affirm whether arginase I operates in this process, we added arginase I or an arginase inhibitor nor-NOHA to CD45^+^CD33^low^CD11b^high^/CD8^+^ T cell or CD45^+^CD33^low^CD11b^dim^/CD8^+^ T cell co-cultures separately. Interestingly, arginase I incubation enhanced CD45^+^CD33^low^CD11b^high^ myeloid cell suppression of CD8^+^ T cell activity in terms of IFN-γ or GrB production (Fig. 3d, e). Additional of nor-NOHA inhibited this suppressive effect (Fig. 3d, e). Clinically, serum arginase I concentration positively correlated with tumor size and tumor stage (Supplementary Figure 3). These results indicated that the CD45^+^CD33^low^CD11b^dim^ myeloid cell subset suppressed CD8^+^ T cell activity at least partly through arginase I production.

### IL-6-induced and IL-8-induced CD45^+^CD33^low^CD11b^dim^ myeloid cell arginase I production via the PI3K-AKT signaling pathway

**Fig. 4:**
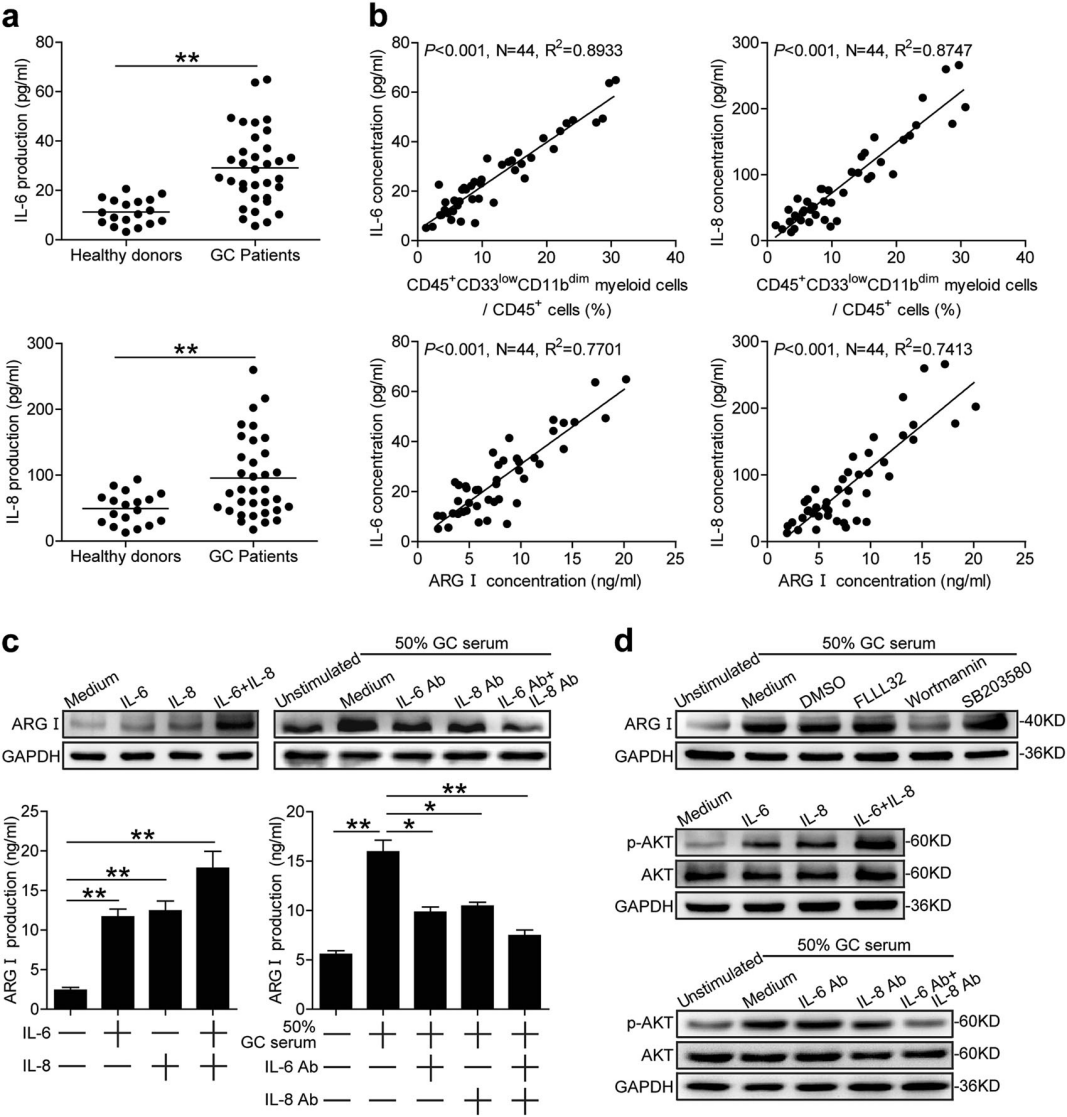
IL-6 and IL-8 induces CD45^+^CD33^low^CD11b^dim^ myeloid cell production of arginase I via PI3K-AKT signaling. **a** Serum IL-6 and IL-8 concentration levels in GC patients compared to healthy donors. **b** Correlations between the proportions of peripheral blood CD45^+^CD33^low^CD11b^dim^ myeloid cells or serum arginase I concentration and IL-6 or IL-8 serum concentrations in GC patients. **c** Arginase I expression and production in CD33^low^CD11b^dim^ myeloid cells exposed to recombinant IL-6 and/or IL-8, or 50% GC patient serum in the presence or absence of IL-6 and/or IL-8 neutralizing abs as analyzed by western blot and ELISA, respectively. **d** Arginase I expression in CD45^+^CD33^low^CD11b^dim^ myeloid cells exposed to 50% GC patient serum in the presence or absence of FLLL32 (STAT3 phosphorylation inhibitor), Wortmannin (PI3K inhibitor), or SB203580 (MAPK inhibitor; top panel). AKT and p-AKT expression levels in CD45^+^CD33^low^CD11b^dim^ myeloid cells exposed to IL-6 and/or IL-8 (middle panel), or 50% GC patient serum in the presence or absence of IL-6 and/or IL-8 neutralizing abs (bottom panels) as analyzed by western blot. *p < 0.05; **p < 0.01, and n.s, *p* > 0.05. ARG I arginase I

IL-6 and IL-8 have been reported to promote the production of arginase I in neutrophils^14,15,18^. We therefore first measured serum levels of IL-6 and IL-8 in GC patients, and found that it was increased compared to healthy donors (Fig. 4a). Serum IL-6 and IL-8 levels positively correlated with GC tumor size and tumor stage (Supplementary Figure 4 and 5). We also found a positive correlation between serum IL-6 and IL-8 levels and the proportion of peripheral blood CD45^+^CD33^low^CD11b^dim^ myeloid cells (Fig. 4b). A positive correlation also existed between serum IL-6 or IL-8 levels and serum arginase I levels in GC patients (Fig. 4b). We next investigated whether GC patient derived serum IL-6 and/or IL-8 could stimulate the production and secretion of arginase I in CD45^+^CD33^low^CD11b^dim^ myeloid cells. To test this, we added recombinant human IL-6 (rhIL-6) and/or IL-8 (rhIL-8) to CD45^+^CD33^low^CD11b^dim^ myeloid cells and incubated for 24 h. Increased arginase I production was detected upon rhIL-6 and/or rhIL-8 addition (Fig. 4c). Arginase I levels conversely decreased in a reciprocal experiment in which neutralizing anti-IL-6 and IL-8 Abs were added to the cultured CD45^+^CD33^low^CD11b^dim^ myeloid cells in the presence of 50% GC patient serum (Fig. 4c).

To investigate which signaling pathway(s) could mediate CD45^+^CD33^low^CD11b^dim^ myeloid cell production of arginase I, we pretreated CD45^+^CD33^low^CD11b^dim^ myeloid cells with a range of MDSC signaling pathway inhibitors before incubation in 50% GC patient serum. Interestingly, decreased arginase I production was observed following pretreatment with the PI3K inhibitor Wortmannin (Fig. 4d). AKT (Ser473) phosphorylation was increased when CD45^+^CD33^low^CD11b^dim^ myeloid cells were stimulated with rhIL-6 and rhIL-8 (Fig. 4d), and decreased following the addition of neutralizing anti-IL-6 and IL-8 Abs to 50% GC patient serum (Fig. 4d). Altogether, these results indicate that IL-6 and IL-8 can induce CD45^+^CD33^low^CD11b^dim^ myeloid cells to increase arginase I production via the PI3K-AKT signaling pathway.

### Blockade of the IL-6/IL-8-arginase I pathway in CD45^+^CD33^low^CD11b^dim^ myeloid cells reduces the degree of CD8^+^ T cell suppression in vitro

**Fig. 5:**
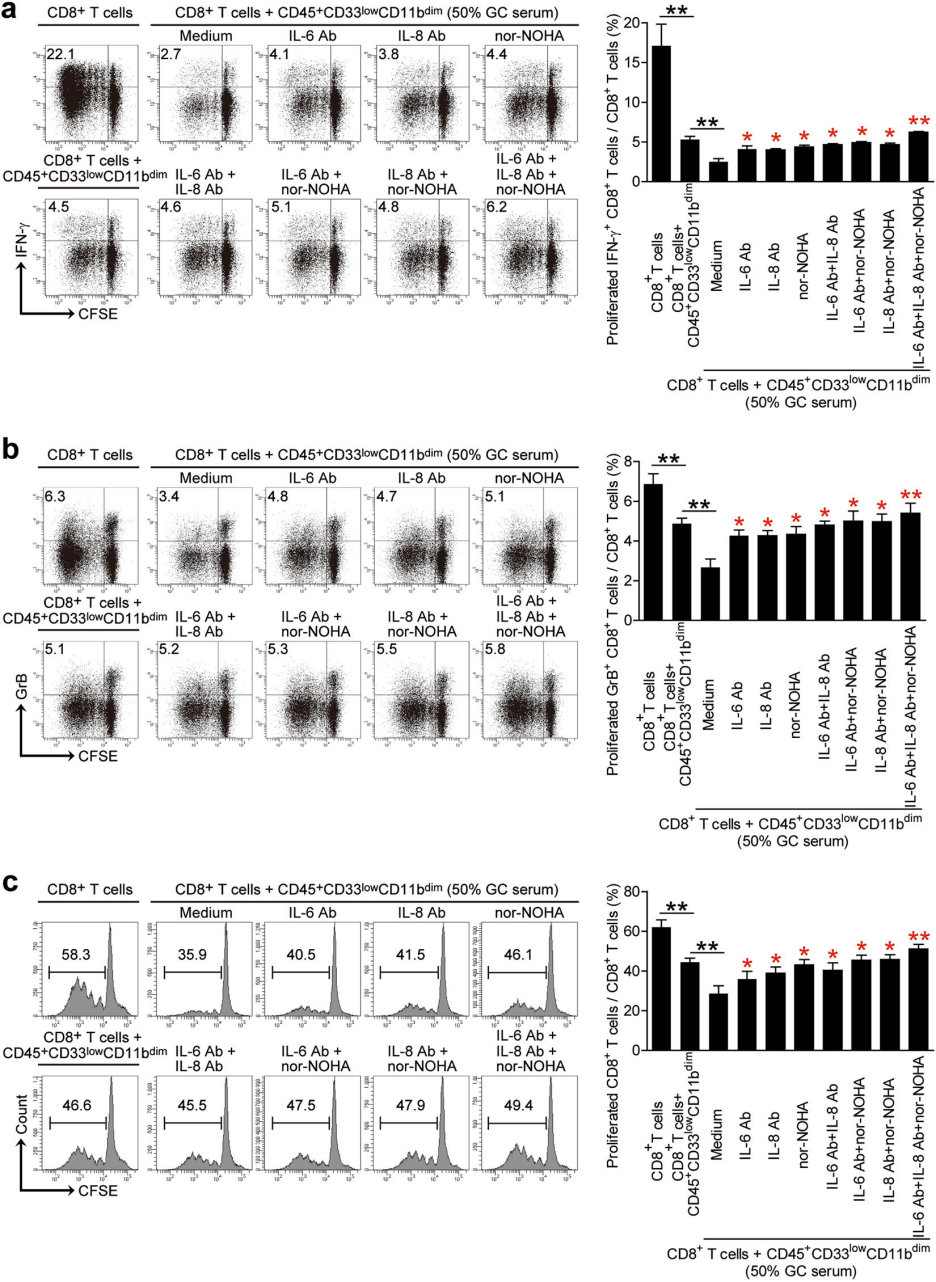
IL-6/IL-8-arginase I blockade reduces CD45^+^CD33^low^CD11b^dim^ myeloid cell-mediated CD8^+^ T cell suppression. CFSE-labeled peripheral blood CD8^+^ T cells from healthy donors were co-cultured for 5 days with peripheral blood CD45^+^CD33^low^CD11b^dim^ myeloid cells from GC patients in the presence or absence of 50% GC patient serum. A proportion of samples were either co-cultured in the presence or absence of IL-6 and/or IL-8 neutralizing abs and/or nor-NOHA, and the proportion of **a** IFN-γ expressing, **b** granzyme B expressing and **c** proliferating CD8^+^ T cells quantitated by flow cytometry. Data representative of three independent experiments (*n* = 3). *p < 0.05; **p < 0.01, and n.s, *p* > 0.05 between respective groups (black) or compared with media control (red)

Neutralizing anti-IL-6 and/or IL-8 Abs and an arginase I inhibitor were added separately or altogether following 50% GC patient serum addition to the CD45^+^CD33^low^CD11b^dim^ myeloid and CD8^+^ T cell co-cultures. Addition of CD45^+^CD33^low^CD11b^dim^ myeloid cells alone or the presence of 50% GC patient serum in the co-culture decreased CD8^+^ T cells proliferation and IFN-γ or GrB production (Fig. 5a–c). Blockade of the IL-6/IL-8-arginase I axis attenuated the degree of reduced CD8^+^ T cell proliferation and IFN-γ or GrB production (Fig. 5a–c). These findings further confirm the involvement of the IL-6/IL-8-arginase I axis in CD45^+^CD33^low^CD11b^dim^ myeloid cell-mediated CD8^+^ T cell suppression.

### CD45^+^CD33^low^CD11b^dim^ myeloid cell frequency correlates with increased tumor progression and decreased patient survival

**Fig. 6:**
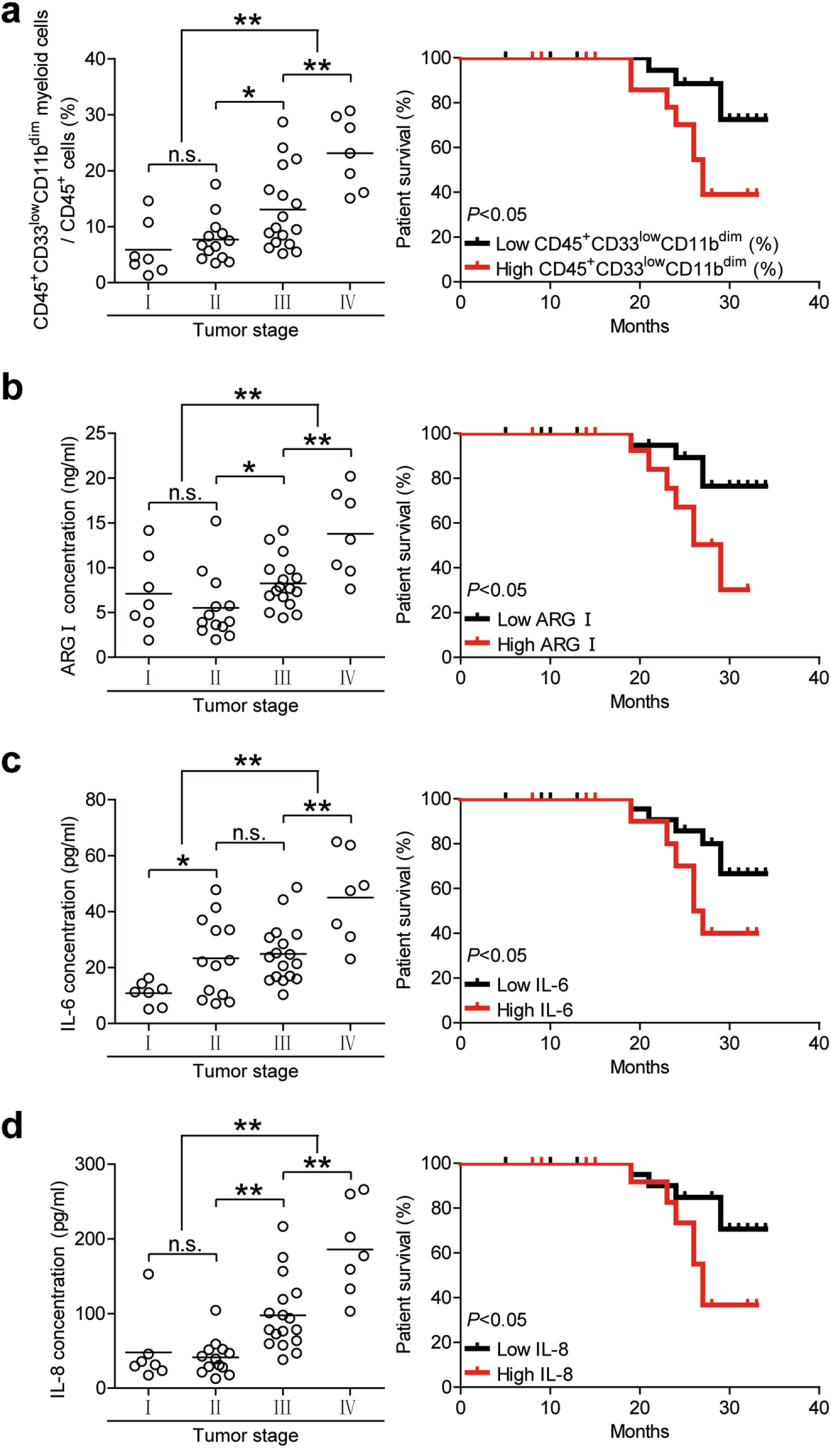
Peripheral blood CD45^+^CD33^low^CD11b^dim^ myeloid cell frequency, and IL-6, IL-8, and arginase I levels correlate with GC tumor progression and decreased patient survival. Peripheral blood (**a**) CD45^+^CD33^low^CD11b^dim^ myeloid cell percentages (**b**) arginase I concentration, (**c**) IL-6 concentration, and (**d**) IL-8 concentration were measured and categorized by patient TNM stage. Kaplan-Meier plots were calculated for overall patient survival according to (**a**) median peripheral blood CD45^+^CD33^low^CD11b^dim^ myeloid cell percentage (9.2%) or (**b**) median arginase I concentration (7.52 ng/ml) or (**c**) median IL-6 concentration (22.37 pg/ml) or (**d**) median IL-8 concentration (61.61 pg/ml). *p < 0.05; **p < 0.01; n.s, *p* > 0.05. ARG arginase I

To further investigate whether CD45^+^CD33^low^CD11b^dim^ myeloid cells were a clinically relevant biomarker of GC, we staged GC patients according to the TNM classification system, and found that peripheral blood CD45^+^CD33^low^CD11b^dim^ myeloid cell frequency was positively associated with advanced stage GC progression (Fig. 6a). A comparison of GC patients based on high (≥9.2% median level) versus low (<9.2%) CD45^+^CD33^low^CD11b^dim^ myeloid cell frequencies showed that the 35-month survival rate was significantly lower in patients with a higher CD45^+^CD33^low^CD11b^dim^ myeloid cell frequency (Fig. 6a). A similar trend was observed when GC patients were separated by high (≥7.52 ng/ml) or low (<7.52 ng/ml) serum arginase I levels (Fig. 6b). We also evaluated the prognostic value of serum IL-6 and IL-8 levels for GC patient survival. The 35-month survival rate was also significantly lower in patients with higher IL-6 (≥ 22.37 pg/ml) or IL-8 (≥ 61.61 pg/ml) serum levels (Fig. 6c, d). Altogether, our findings demonstrate that peripheral blood CD45^+^CD33^low^CD11b^dim^ MDSCs suppress CD8^+^ T cell activity via the IL-6/IL-8-arginase I axis and are positively associated with GC tumor progression and negatively associated with overall patient survival.

## Discussion

MDSCs are a phenotypically and functionally heterogeneous population categorized by their ability to functionally suppress T cell activity in cancer patients and tumor-bearing mice^6,10^. In mice, MDSCs are phenotypically CD11b^+^Gr1^+^ like neutrophils and monocytes during homeostasis and classified as either polymorphonuclear (PMN)-MDSCs or monocytic (MO)-MDSCs. Murine PMN-MDSCs suppressed CD8^+^ antigen-specific T cell activity via ROS production, while MO-MDSCs suppressed CD8^+^ T cell activity non-specifically through arginase I and NO production^19–21^. In humans, MDSCs are also classified into PMN-MDSCs or MO-MDSCs. The neutrophil marker CD66b, a member of the carcinoembryonic antigen-like glycoprotein family, has also been reported as a marker of MDSCs in patients with head and neck cancer^22^, renal cell carcinoma^23^, and autoimmune diseases such as systemic lupus erythematosus^24^. In GC patients, we identified CD45^+^CD11b^+^CD33^+^ classical MDSCs as well as two additional subsets of myeloid cells, one is CD45^+^CD33^low^CD11b^dim^ and the other is CD45^+^CD33^low^CD11^high^. However, in the peripheral blood of healthy donors, we only identified CD45^+^CD33^low^CD11^high^ cells (Fig. 1a). We therefore suspect that the appearance of CD45^+^CD33^low^CD11b^dim^ cells may be associated with GC. We then compared the functions of the two cell populations. Both CD45^+^CD33^low^CD11b^dim^ cells and CD45^+^CD33^low^CD11^high^ cells suppressed CD8^+^ T cell activity, although the CD45^+^CD33^low^CD11b^dim^ cells showed a stronger suppress activity (Fig. 2). These results indicated that both subsets function as myeloid suppressor cells in the circulation. It is possible that the CD45^+^ CD33^low^CD11^high^ cells may suppress autoantigen reactive T cells in healthy donors and play a role in preventing or limiting autoimmune diseases; however the CD45^+^CD33^low^CD11b^dim^ cells may function in the peripheral blood of GC patients to promote an immunosuppressive tumor microenvironment. We therefore focused on CD45^+^CD33^low^CD11b^dim^ MDSCs.

CD45^+^CD33^low^CD11b^dim^ MDSCs express CD66b, they also possess multi-lobar nuclei, further indicative of a granulocytic rather than MO phenotype^22,23^(Fig. 1c, d). Since CD45^+^CD33^low^CD11b^dim^ MDSCs were detectable in peripheral blood from GC patients but healthy donors, it is likely for them to have been selectively expanded by factors derived from the GC microenvironment. In lieu with this, we found that CD45^+^CD33^low^CD11b^dim^ MDSCs significantly suppressed CD8^+^ T cell activity in vitro and also correlated positively in frequency with GC stage and negatively with overall patient survival (Fig. 6). To our knowledge, this is the first description of a CD45^+^CD33^low^CD11b^dim^ subset of PMN-MDSCs in GC patients.

Aside from phagocytosis, mature granulocytes primarily exert their functions through the release of primary, secondary, and tertiary granules. We therefore speculated whether CD8^+^ T cell suppression by CD45^+^CD33^low^CD11b^dim^ MDSCs was dependent on the secretion of soluble factors, similar to classical MDSC subsets. MDSCs are recruited by pro-inflammatory signals from the tumor microenvironment or during autoimmune disease and other pathologic conditions. Functionally, they exert their immunosuppressive activities through the upregulation of arginase I, iNOS, IDO, NO, and ROS^9,10^. Since MDSC frequency positively correlates with serum arginase I levels^24^ and arginase I is constitutively expressed in human granulocytes^25^, we hypothesized whether granulocytic CD45^+^CD33^low^CD11b^dim^ MDSCs could suppress CD8^+^ T cells via arginase I production. A positive correlation was found between serum arginase I levels and the frequency of peripheral blood CD45^+^CD33^low^CD11b^dim^ MDSCs in our cohort of GC patients, serum arginase I levels were increased in GC patients compared to healthy donors (Fig. 3a). Arginase I metabolizes L-arginine from the extracellular environment^11^. L-arginine depletion by MDSCs has been reported to inhibit the re-expression of the CD3 TCRζ-chain and impair antigen-specific CD8^+^ and CD4^+^ T cell proliferation^12^. CD45^+^CD33^low^CD11b^dim^ MDSCs also inhibited CD8^+^ T cell proliferation and IFN-γ or GrB production in vitro, in accordance with MDSC-mediated immunosuppression. Granzymes are stored in granules in cytotoxic lymphocytes^26,27^ and released upon stimulation^28,29^. However, production of IFN-γ starts from the activation of STAT1 and T-bet, through transcription and translation, which takes 8–11 h to reach the peak^30^. Therefore the production of IFN-γ by CD45^+^CD33^low^CD11b^dim^ MDSCs after treatment was likely not yet completely restored when compared with granzymes (Fig. 5).

Numerous serum or tumor-derived pro-inflammatory factors have been implicated in MDSC survival and/or activity. Increased levels of serum IL-6 and IL-8 are commonly observed in cancer patients, and are positively correlated with the number of infiltrating MDSCs in breast cancer^31^. IL-6 induces neutrophil elastase release^32^, while IL-8 induces neutrophil exocytosis of arginase I in non-small cell lung cancer patients^15^. In our study, we also found a positive correlation between serum IL-6 and IL-8 concentrations and the frequency of peripheral blood CD45^+^CD33^low^CD11b^dim^ MDSCs in GC patients (Fig. 4a). IL-6 and IL-8 serum concentrations also positively correlated with the concentration of serum arginase I in GC patients (Fig. 4a). In our study, GC patient derived-serum IL-6 and IL-8 both stimulated arginase I production and release in CD45^+^CD33^low^CD11b^dim^ MDSCs. Besides, the gene expression profile of human PMN-MDSCs includes mTOR, MAPK, and other related signaling pathways^13,33^. We found that the activation of CD45^+^CD33^low^CD11b^dim^ MDSCs were through the PI3K/Akt pathway (Fig. 4d), which has crosstalk with mTOR signaling pathway, and may also induce direct activation or phosphorylation of mTOR.

**Fig. 7:**
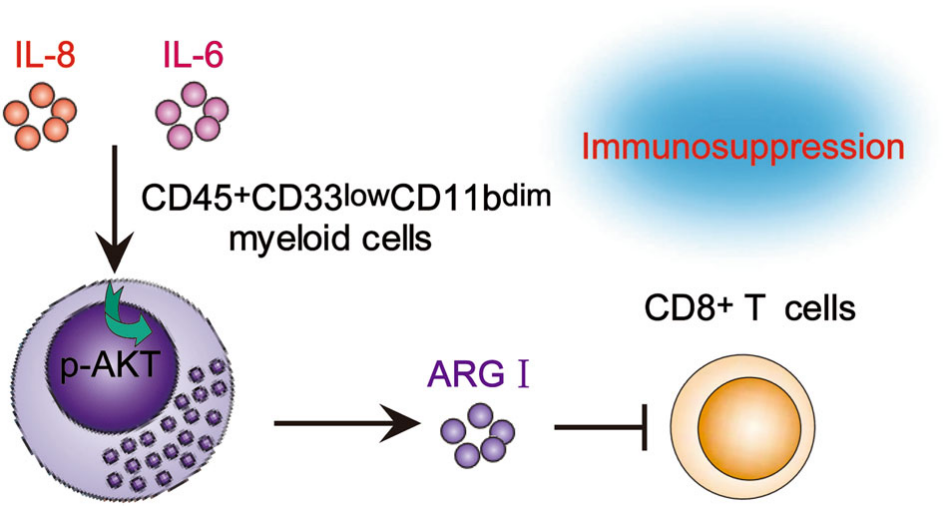
Peripheral blood CD45^+^CD33^low^CD11b^dim^ myeloid cells suppress CD8^+^ T cells through the IL-6/IL-8-arginase I (ARG I) axis in GC patients. Circulating IL-6 and IL-8 induces the secretion of arginase I from CD45^+^CD33^low^CD11b^dim^ myeloid cells via the PI3K-AKT signaling pathway

Our findings indicate a role for the IL-6/IL-8-arginase I axis in CD45^+^CD33^low^CD11b^dim^ MDSCs-mediated CD8^+^ T cell immunosuppression in GC patients (Fig. 7). GC patient serum-derived IL-6 and IL-8 activates the PI3 kinase signaling pathway in CD45^+^CD33^low^CD11b^dim^ MDSCs to induce arginase I production and release, thereby inhibiting the activity of CD8^+^ T cells. Overall, our study re-emphasizes the heterogeneous spectrum of MDSC subsets identified in cancer patients, and suggests that the IL-6/IL-8-arginase I axis is a potential therapeutic target for the ablation of MDSC-mediated immunosuppression in GC patients.

## Materials and methods

### Patients samples

GC patients who underwent surgical resection without prior radiotherapy or chemotherapy treatment between January 2015 and December 2017 were enrolled at the Southwest Hospital of Army Medical University. GC patients with concurrent autoimmune diseases, infectious diseases, or multiple primary cancers were excluded from the study. Peripheral blood samples from 45 healthy volunteers were used as controls. The clinical stages of tumors were determined according to the TNM classification system of International Union Against Cancer (Edition 7). Individual GC patient characteristics are provided in Supplementary Table 1. This study was reviewed and approved by the Ethics Committee of the Southwest Hospital of Army Medical University, and written informed consent for study enrollment and blood collections obtained from all individuals.

### Antibodies and other reagents

Antibodies (Abs) used for flow cytometry were anti-IFN-γ-PE-Cy7 (BD Pharmingen); anti-CD66b-FITC, anti-CD33-PE, anti-CD45-PE-Cy7, anti-CD14-PerCP-Cy5.5, anti-HLA-DR-Alexa Fluor 647 and CD8-PerCP-Cy5.5 (eBioscience); anti-perforin-PE, and anti-granzyme B-APC (Biolegend). Abs used for western blotting were anti-human arginase I (R&D Systems) and horseradish peroxidase (HRP)-conjugated secondary ab (Zhongshan Biotechnology). Abs used for IL-6/IL-8 blockade studies were: anti-human IL-6 (Biolegend) and anti-human IL-8 (R&D Systems). The chemical arginase I inhibitor nor-NOHA (Santa Cruz) was also used. Purified anti-CD3 and anti-CD28 Abs (Biolegend) and recombinant human IL-2 (PeproTech) were also used for T cell studies.

Recombinant human IL-6, IL-8, and arginase I (Biolegend) were used for CD45^+^CD33^low^CD11b^dim^ activation studies. Drug inhibitors studies involved the STAT3 inhibitor FLLL32 (Medco Biosciences), PI3K inhibitor Wortmannin, and MAPK inhibitor SB203580 (Calbiochem).

### Flow cytometry

A 100 µl of peripheral blood was incubated with primary ab cocktails for 30 min at 4 °C in the dark. Erythrocytes were lysed with the FACS lysing solution for 10 min in the dark. Samples were centrifuged for 5 min at 300 g and the supernatant was discarded. The cell pellet was re-suspended in PBS and flow cytometric analysis performed on the BD FACS Canto II using FACS Diva version 6.1.2 (BD Biosciences).

For intracellular cell staining (ICS), cells were stimulated for 5 h with the leukocyte activation cocktail with BD Golgiplug (BD Pharmingen). ICS was performed after cell fixation and permeabilization, using the Perm/Wash solution (BD Pharmingen) as per manufacturer’s protocols. Cells were analyzed using the FACSCanto II with FACS Diva version 6.1.2 (BD Biosciences).

### Fluorescence-activated cell sorting

A 20 ml of peripheral blood was stained with anti-human CD45, anti-human CD33 and anti-human CD11b Ab, and erythrocytes lysed as per above. The cell pellet was then suspended in PBS at a concentration of 5 × 10^6^ cells/ml. CD45^+^CD33^low^CD11b^high^ cells and CD45^+^CD33^low^CD11b^dim^ cells were sorted by FACS using the FACS Aria II (BD Biosciences) and collected in RPMI 1640 containing 10% FCS.

### Myeloid and T cell co-cultures

Peripheral blood mononuclear cells (PBMCs) were isolated by density gradient centrifugation with Ficoll-Paque Plus (GE Healthcare). 10^5^ magnetic bead-purified peripheral CD8^+^ T cells from PBMCs (STEMCELL Technologies) were labeled with carboxyfluoresceinsuccinimidyl ester (CFSE; eBioscience) and cultured in RPMI 1640 containing 10% FCS with recombinant human IL-2 (20 IU/ml), with pre-coated anti-CD3 (2 μg/ml) and anti-CD28 (1 μg/ml) abs on the plate.

CD45^+^CD33^low^CD11b^high^ and CD45^+^CD33^low^CD11b^dim^ myeloid cells were sorted as described above and added with CD8^+^ T cells at a 1:1, 1:2, 1:4, or 1:8 ratio. In some experiments, autologous human serum was added into the co-culture system (50% serum) and/or neutralizing abs against IL-6 (10 μg/ml), IL-8 (10 μg/ml), and arginase I (25 μM) or nor-NOHA (250 μM) also added to the co-culture system. After 5 days incubation, cell pellets resuspended in PBS for flow cytometry.

### Western blot analysis

Total proteins from vehicle control or IL-6, IL-8, GC serum, or drug inhibitor pretreated CD45^+^CD33^low^CD11b^dim^ myeloid cells were extracted with Pierce reagent. Western blot assays were performed on 10–15% SDS-PAGE gels using equivalent amounts of cell lysate proteins per sample. Proteins were transfer onto a PVDF membrane and blocked with 3% BSA. Human arginase I was detected with anti-arginase I abs and human phosphorylated AKT-ser473 or total AKT were detected with anti-p-AKT or anti-AKT abs, followed by incubation with HRP-conjugated secondary abs and visualization using the SuperSignal® West Dura Extended Duration Substrate kit (Thermo Fisher Scientific).

### RNA extraction and quantitative real-time PCR

Total RNA of CD45^+^CD33^low^CD11b^high^ cells and CD45^+^CD33^low^CD11b^dim^ cells were extracted with TRIzol reagent. The RNA quality was measured with NANO2000, and samples were reversed transcript to double-stranded cDNA with PrimeScript^TM^ RT reagent Kit (TaKaRa). Quantitative Real-time PCR was performed using the primers listed in Supplementary Table 2 on the IQ5 (Bio-Rad) with the Real-time PCR Master Mix (Toyobo). Expression of arginase I was normalized against human GAPDH, and the results were calculated by the ΔΔCt method and expressed as fold change.

### ELISA

ELISA was performed on serum or the supernatant of cell cultures. Human IL-6 (eBioscience), IL-8 (eBioscience), IFN-γ (Biolegend) and arginase I (Cusabio Biotech) were detected. The concentration of serum IL-6, IL-8, and arginase I and T cell culture-derived IFN-γ was determined using Enzyme-linked immunosorbent assay (ELISA) kits according to manufacturer’s instructions.

### Statistical analysis

Flow cytometry analysis was performed using Flowjo software or BD FACS Diva software. All results are displayedas mean ± SEM, with each data point representing individual patients or patient samples. Statistical analysis performed with the SPSS statistical software (version 13.0). The Student *t* test or Mann-Whitney *U* test was used to analyze the difference between the mean of two groups depending on the nature of sample variance. Pearson correlation analysis and linear regression analysis were used to calculate correlations. Overall patient survival was defined as the interval between the date of surgery and date of death or last clinical follow-up, whichever occurred earlier. Cumulative survival time was calculated by the Kaplan-Meier method, with survival measured in months and the log-rank test applied for comparisons between two groups. A two-sided *p* value of <0.05 was considered statistically significant.

## Electronic supplementary material

supplementary figure legends

supplementary table 1

supplementary table 2

supplementary figure 1

supplementary figure 2

supplementary figure 3

supplementary figure 4

supplementary figure 5
