# Supplementary material for: CD45+CD33lowCD11bdim myeloid-derived suppressor cells suppress CD8+ T cell activity via the IL-6/IL-8-arginase I axis in human gastric cancer
Source: Cell Death Dis. 2018 Jul 9;9(7):763. doi: 10.1038/s41419-018-0803-7 (PMC6037756; doi:10.1038/s41419-018-0803-7)
Supplement: Supplementary file 1 — supplementary figure legends [file 41419_2018_803_MOESM1_ESM.doc]

Supplementary Figure 1. Expression of neutrophil marker CD66b on circulating CD45^+^CD33^low^CD11b^high^ myeloid cells and expression of monocyte marker CD14 on circulating CD45^+^CD33^high^CD11b^high^ myeloid cells by gating on CD45^+^ cells in blood from GC patients.

Supplementary Figure 2. Circulating CD45^+^CD33^low^CD11b^dim^ myeloid cell percentage and its potential correlations with clinical parameters. Circulating CD45^+^CD33^low^CD11b^dim^ myeloid cell percentage in CD45^+^ leukocytes was analyzed for correlations with clinical pathological parameters. *, *p*<0.05; **, *p*<0.01, and n.s, *p*>0.05 for groups connected by horizontal lines. Each dot represents one patient. CEA, carcinoembryonic antigen; *H.pylori* Ab, *Helicobacter pylori* antibody.

Supplementary Figure 3. The concentration of arginase Ⅰ and its potential correlations with clinical parameters. The concentration of arginase Ⅰ in serum was analyzed for correlations with clinical pathological parameters. *, *p*<0.05; **, *p*<0.01, and n.s, *p*>0.05 for groups connected by horizontal lines. Each dot represents one patient. CEA, carcinoembryonic antigen; *H.pylori* Ab, *Helicobacter pylori* antibody.

Supplementary Figure 4. The concentration of IL-6 and its potential correlations with clinical parameters. The concentration of IL-6 in serum was analyzed for correlations with clinical pathological parameters. *, *p*<0.05; **, *p*<0.01, and n.s, *p*>0.05 for groups connected by horizontal lines. Each dot represents one patient. CEA, carcinoembryonic antigen; *H.pylori* Ab, *Helicobacter pylori* antibody.

Supplementary Figure 5. The concentration of IL-8 and its potential correlations with clinical parameters. The concentration of IL-8 in serum was analyzed for correlations with clinical pathological parameters. *, *p*<0.05; **, *p*<0.01, and n.s, *p*>0.05 for groups connected by horizontal lines. Each dot represents one patient. CEA, carcinoembryonic antigen; *H.pylori* Ab, *Helicobacter pylori* antibody.
