# Supplementary material for: CD45+CD33lowCD11bdim myeloid-derived suppressor cells suppress CD8+ T cell activity via the IL-6/IL-8-arginase I axis in human gastric cancer
Source: Cell Death Dis. 2018 Jul 9;9(7):763. doi: 10.1038/s41419-018-0803-7 (PMC6037756; doi:10.1038/s41419-018-0803-7)
Supplement: Supplementary file 2 — supplementary table 1 [file 41419_2018_803_MOESM2_ESM.doc]

**Supplementary Table 2.** Clinical characteristics of 44 patients with gastric cancer

| Variables | No. of patients |
| --- | --- |
| Gender (male/female) | 28/15 |
| Age (years; median, range) | 54, 34-82 |
| *H.pylori* Ab (negative/positive) | 12/32 |
| CEA (U/L; <5/≥5) | 28/16 |
| Tumor size (cm; <5/≥5) | 28/16 |
| Lymphatic invasion (absent/present) | 18/26 |
| Vascular invasion (absent/present) | 33/11 |
| Tumor (T) invasion (T1+T2/T3+T4) | 20/24 |
| Lymphoid Nodal (N) status (N0+N1/N2+N3) | 24/20 |
| Distant metastasis (M) status (M0/M1) | 38/6 |
| TNM stage (Ⅰ+Ⅱ/Ⅲ+Ⅳ) | 20/24 |

CEA, carcinoembryonic antigen; *H.pylori* Ab, *Helicobacter pylori* antibody.
