# Supplementary material for: CD45+CD33lowCD11bdim myeloid-derived suppressor cells suppress CD8+ T cell activity via the IL-6/IL-8-arginase I axis in human gastric cancer
Source: Cell Death Dis. 2018 Jul 9;9(7):763. doi: 10.1038/s41419-018-0803-7 (PMC6037756; doi:10.1038/s41419-018-0803-7)
Supplement: Supplementary file 3 — supplementary table 2 [file 41419_2018_803_MOESM3_ESM.doc]

**Supplementary Table 2. Primer and probe sequences for real-time PCR analysis**

| Gene | Primer or probe | Sequence 5′→3′ |
| --- | --- | --- |
| Human Arginase I | forward | GGCTGGTCTGCTTGAGAAAC |
|  | reverse | ATTGCCAAACTGTGGTCTCC |
| Human GAPDH | forward  reverse | ACCCAGAAGACTGTGGATGG  CAGTGAGCTTCCCGTTCAG |

For the probes, a FAM fluorescent reporter is coupled to the 5' end, and a TAMRA quencher is coupled to the 3' end.
