# Supplementary figures and images for: CD45+CD33lowCD11bdim myeloid-derived suppressor cells suppress CD8+ T cell activity via the IL-6/IL-8-arginase I axis in human gastric cancer
Source: Cell Death Dis. 2018 Jul 9;9(7):763. doi: 10.1038/s41419-018-0803-7 (PMC6037756; doi:10.1038/s41419-018-0803-7)

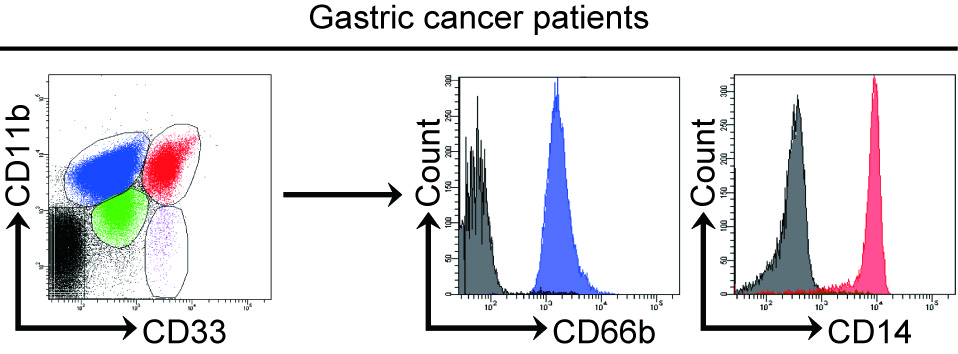

Supplement: Supplementary file 4 — supplementary figure 1 [file 41419_2018_803_MOESM4_ESM.jpg]

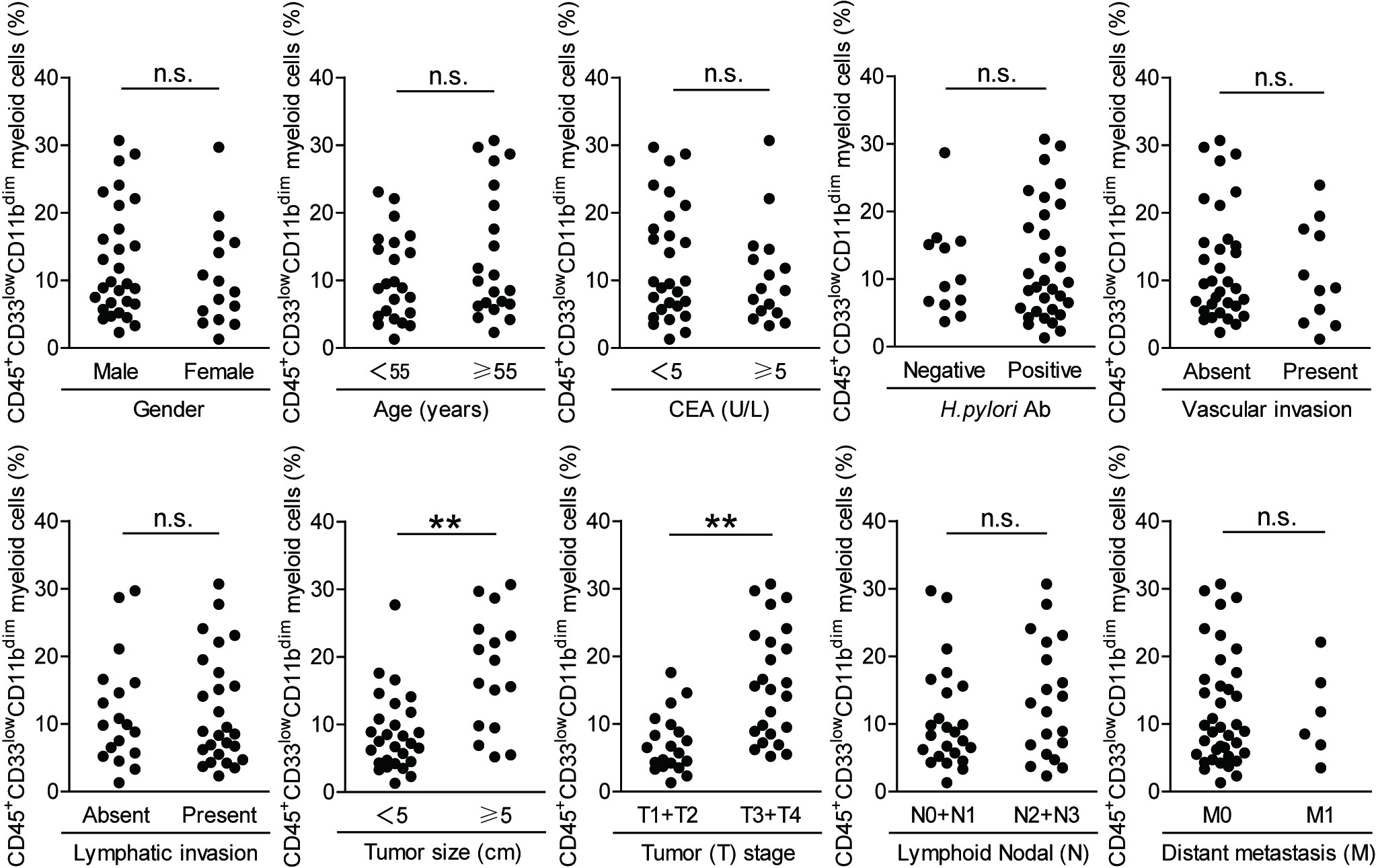

Supplement: Supplementary file 5 — supplementary figure 2 [file 41419_2018_803_MOESM5_ESM.jpg]

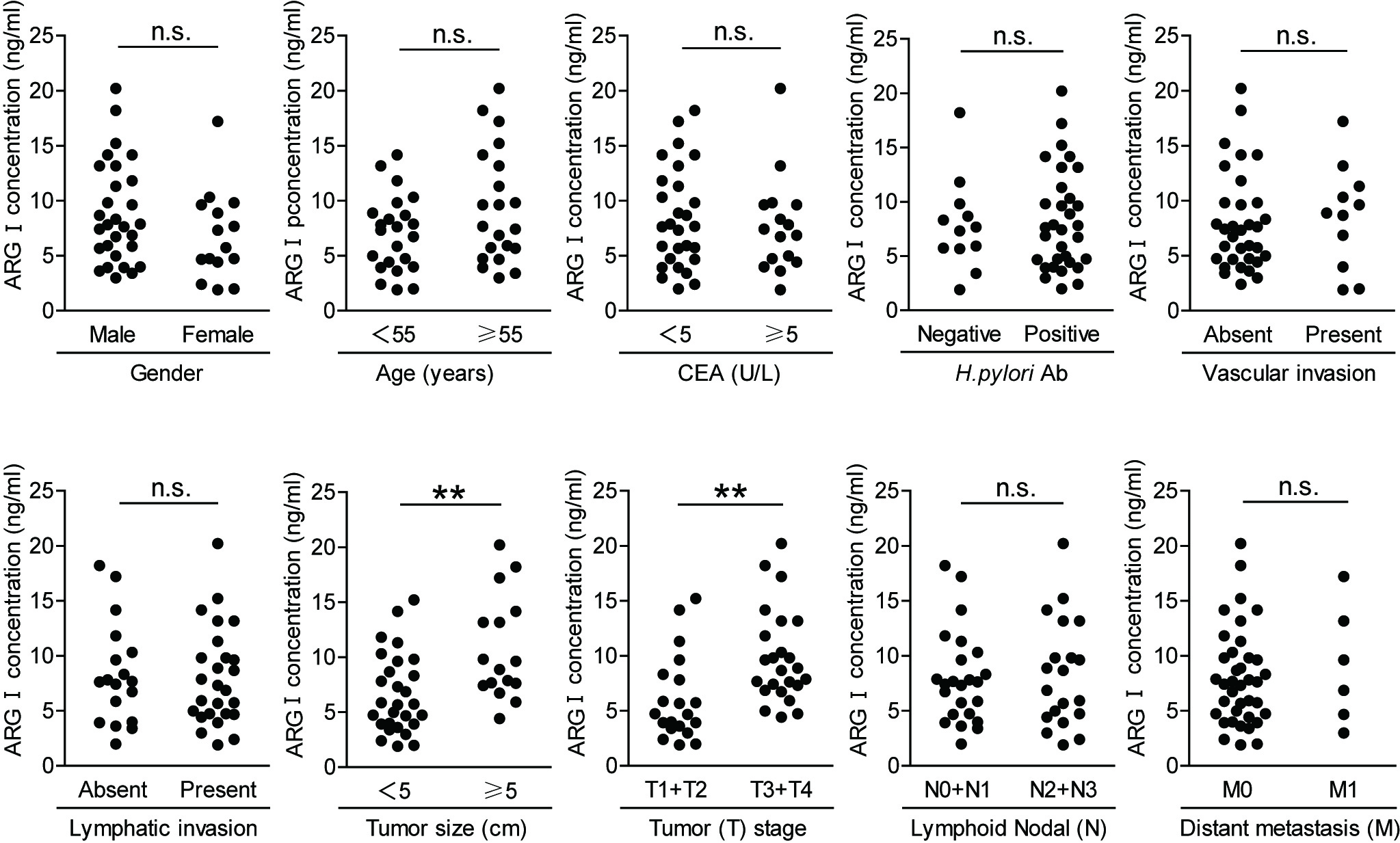

Supplement: Supplementary file 6 — supplementary figure 3 [file 41419_2018_803_MOESM6_ESM.jpg]

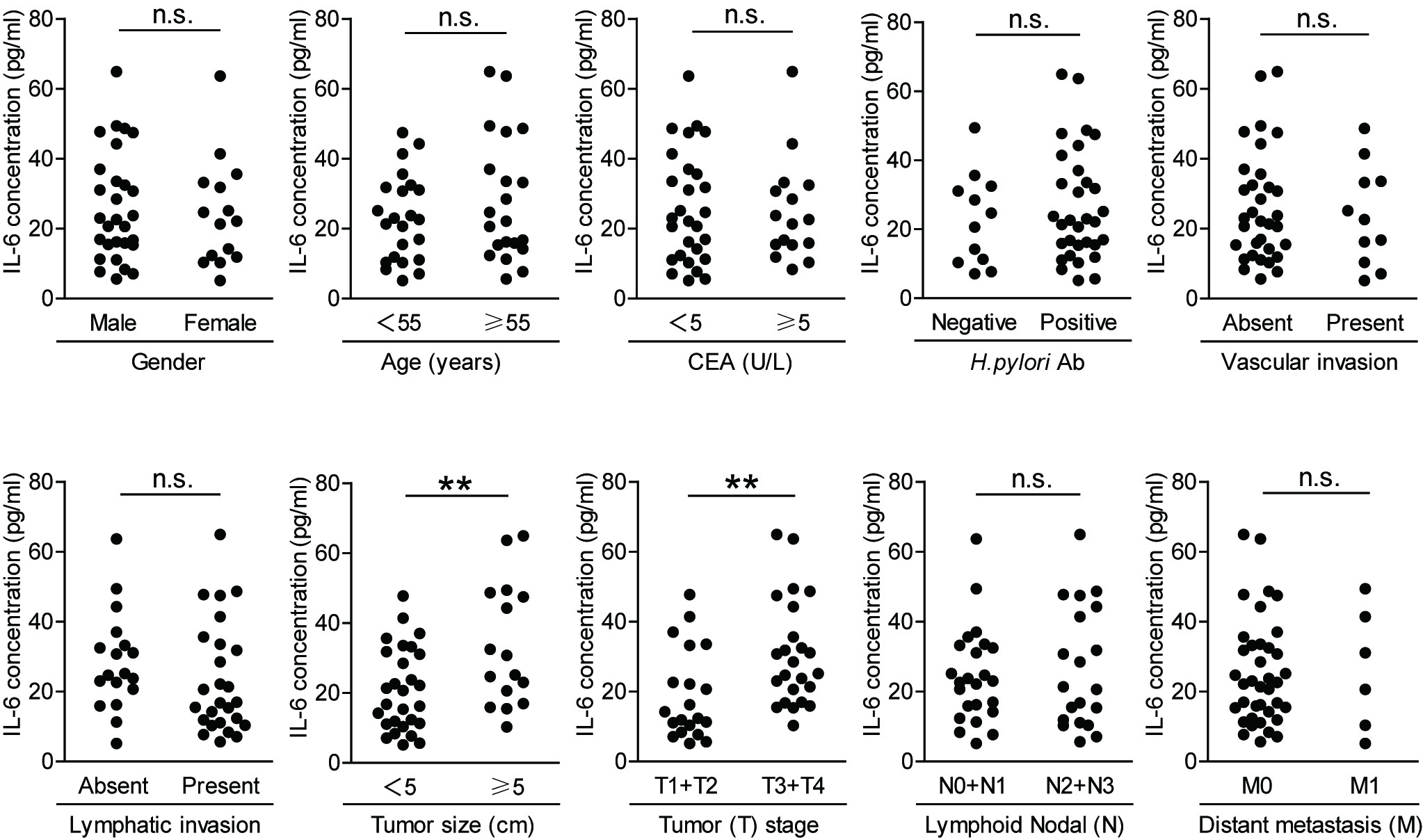

Supplement: Supplementary file 7 — supplementary figure 4 [file 41419_2018_803_MOESM7_ESM.jpg]

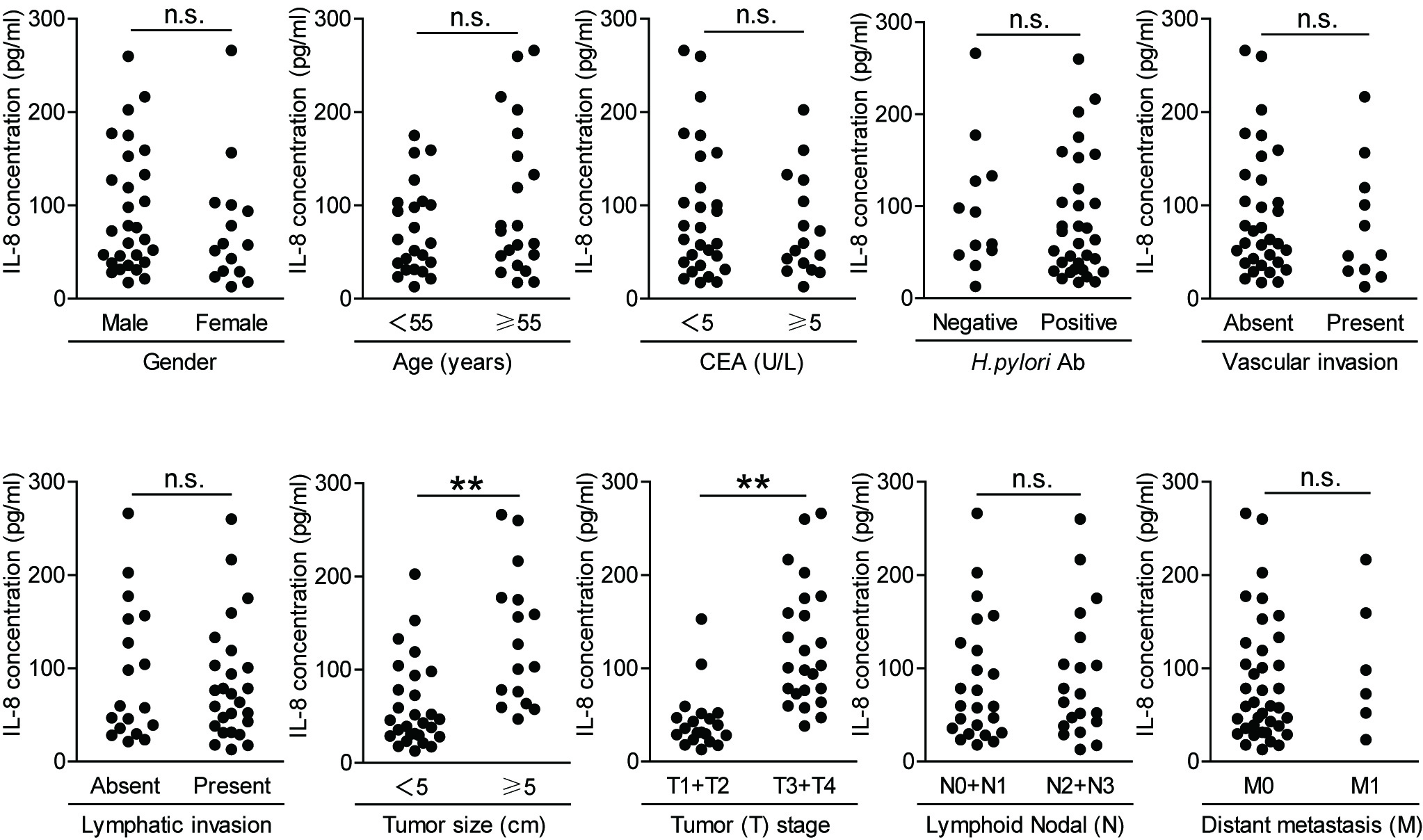

Supplement: Supplementary file 8 — supplementary figure 5 [file 41419_2018_803_MOESM8_ESM.jpg]
